# *Arthrospira platensis* Mitigates LPA-Induced Endothelial Dysfunction: A Prospective, Placebo-Controlled Study

**DOI:** 10.3390/cells15080694

**Published:** 2026-04-14

**Authors:** Anne Krüger-Genge, Conrad Jung, Sophia Westphal, Kudor Harb, Joachim Storsberg, Steffen Braune, Jan-Heiner Küpper, Friedrich Jung

**Affiliations:** 1 Life Science and Bioprocesses, Fraunhofer-Institute for Applied Polymer Research (IAP), 14469 Potsdam, Germany; sophia.westphal@iap.fraunhofer.de (S.W.); kudor.harb@charite.de (K.H.); joachim.storsberg@iap.fraunhofer.de (J.S.); 2 Carbon Biotech, Social Enterprise Stiftungs AG, 01968 Senftenberg, Germany; c.jung@carbonbiotech.eu (C.J.); jan-heiner.kuepper@b-tu.de (J.-H.K.); 3 Faculty of Medicine, Private University in the Principality of Liechtenstein (UFL), 9495 Triesen, Liechtenstein; 4 Institute of Biotechnology, Molecular Cell Biology, Brandenburg University of Technology, 01968 Senftenberg, Germany; steffen.braune@b-tu.de (S.B.); friedrich.jung@b-tu.de (F.J.)

**Keywords:** *Arthrospira platensis*, lipopolysaccharide, primary human venous endothelial cells, LPA

## Abstract

**Highlights:**

**What are the main findings:**

- *Arthrospira platensis* extracts mitigate LPA-induced endothelial dysfunction.
- A concentration of 100 ug/mL of *Arthrospira platensis* was found to be the most effective dose.

**What are the implications of the main findings?**

- *Arthrospira platensis* has the potential to reduce venous endothelium (proofed in vitro) damage when exposed to LPA as component of lipopolysaccharide.
- *Arthrospira platensis* has a potential therapeutic role in conditions associated with endothelium dysfunction, such assepsis.

**Abstract:**

(1): Endotoxins are components of Gram-negative bacteria. Uptake can induce allergies, nausea, or sepsis. These responses are triggered by an activation of the immune system. Endothelial cells, lining blood vessels, are the first to be exposed to circulating LPA. Activation can dramatically affect the blood system, such as the formation of thrombi. This study aimed to clarify whether the activation of primary human venous endothelial cells (HUVECs) by LPA could be reduced by the addition of an *Arthrospira platensis* (AP) extract. (2): HUVECs were cultured for 24 h in cell culture medium supplemented with different concentrations of AP (50, 100, 200 µg/mL). Then 2.5 µg/mL of LPA was added. Cell morphology, viability, cell proliferation, cell membrane integrity, cell metabolism, and cell function were examined after two and four days. (3): Treatment with LPA alone negatively affected HUVEC growth, viability, cell membrane integrity, and metabolic activity. Adding AP to the culture medium had a positive influence on these effects, with 100 µg/mL proving to be the most effective dose. (4): The results clearly revealed that an extract of AP has the potential to reduce the damage to the venous endothelium when exposed to lipopolysaccharides, in particular at a concentration of 100 µg/mL.

## 1. Introduction

Aerosols from waste, natural raw materials, agriculture and animal feed, storage and forestry may contain endotoxins. Endotoxins can also be released from bacteria-colonized water by (e.g., in humidifiers, cooling systems, wastewater or sewage treatment plants). One such endotoxin is lipopolysaccharide (LPS), which is found in the outer membrane of Gram-negative bacteria [1]. LPS from Gram-negative microbes does not necessarily require the disruption of the bacterial cell wall, but rather, LPS is released as part of the physiological activity of membrane vesicle trafficking [2,3]. LPS consists of three components: a core oligosaccharide, an O-specific chain consisting of repeated polysaccharide sequences, and lipid A (LPA), which is responsible for the proinflammatory nature of LPS [4]. Endothelial cells (EC) are located on the inner wall of blood vessels and are therefore the first cells, after circulating blood cells, to be exposed to invading pathogens such as LPS circulating in the bloodstream [5]. LPS is released from the surface of replicating and dying Gram-negative bacteria into the bloodstream, where it can then interact with the endothelium [6]. At the molecular level, LPS binds to LPS-binding protein (LBP) and is transported to a complex of the Toll-like receptor 4 (TLR4), myeloid differentiation factor 2 (MD-2) and cluster of differentiation 14 (CD14) [7]. Binding of LPS to TLR4 results in endothelial perturbation, which is associated with the expression of proinflammatory cytokines and adhesion molecules and in severe cases beyond that with endothelial apoptosis [8,9,10,11,12]. Several studies have shown that LPS can also activate macrophages and neutrophils, resulting in the secretion of various interleukins (IL-1, IL-6, IL-8), tumor necrosis factor (TNFα) and interferons (IHNs) as well as activating the classic and alternative complement pathways and the arachidonic acid cycle [13]. In addition to circulating blood cells, vascular endothelial cells represent the main target cells for LPS and play a key role in the development and formation of thrombi [14,15,16]. In septic-like conditions, caused by LPS infection, microthrombosis with subsequent organ failure may occur [17,18,19]. While it is well known that LPS-induced endotoxemia accelerates thrombus formation in the microcirculation in vivo [20], effects on thrombosis in large arteries or veins have also been described [21].

A first pilot study revealed that supplementation of HUVEC culture medium with increasing concentrations of LPS (5, 25 and 50 μg/mL) resulted in a progressive reduction in the density of adherent HUVEC after shear stress (*p* < 0.001) [9]. 48% of the HUVEC in control cultures (0 μg/mL LPS) were still adherent after 2 h of shearing at 6 dyne/cm^2^, whereas 80 min after the addition of 50 μg/mL LPS, 88% of the HUVEC had already detached from the substrate and after 100 min no more HUVEC were adherent. Also, under static conditions, a preliminary study showed that the cell index (as a measure of the density of an endothelial cell monolayer) measured continuously over 80 h decreased significantly after LPS addition compared to control cultures [22]. This result could now be confirmed in a prospective, blinded, placebo-controlled and sample size-estimated in vitro study [23].

*Arthrospira platensis* (AP) is a blue–green, filamentous, photosynthetic, multicellular cyanobacterium characterized by helicoidal trichomes [24]. AP is used as food for animals and humans and in the human health food industry for its therapeutic properties, including antioxidant, anti-inflammatory, immunomodulatory and anticancer activities [25,26,27,28,29]. In addition, a protective effect of AP against various chemical and biological pathogens has been reported [30,31,32].

The aim of this study was therefore to investigate whether AP can reduce LPA-induced dysfunction of human venous endothelial cells as component of LPS, a known strong endotoxin, and which cellular and molecular changes in the cells are associated with this.

## 2. Materials and Methods

### 2.1. Study Design

The study was performed using a prospective, controlled design and primary human umbilical vein endothelial cells (HUVEC). In vitro cell culture experiments were performed following procedures described earlier.

At baseline 15,000 HUVEC were seeded per well and the formation of a HUVEC monolayer on tissue culture plates (TCP) was evaluated for over 80 h. All tests were performed with negative controls (vehicle solution/0.9% NaCl) and positive controls (lysed HUVEC). All experiments were performed with eight replicates according to a data-based sample size estimation.

After seeding HUVECs in 24-well plates the cell culture medium was supplemented with an aqueous *Arthrospira platensis* (AP) extract at three different concentrations (50 µg/mL, 100 µg/mL, 200 µg/mL). These concentrations were chosen because prior studies in the concentration range von 50–1000 µg/mL have shown (unpublished data), that in the range between 50 and 200 µg/mL an increased proliferation rate of HUVEC was found.

After 24 h, LPA (2.5 µg/mL, Sigma-Aldrich, St. Louis, MO, USA) was added to the culture medium. This was done to induce potential HUVEC damage.

The following cellular parameters were investigated: cell adherence and proliferation, cell viability, cell membrane integrity, cell metabolism, cell function and cell–cell contact, and stress fiber formation. HUVECs in a pure cell culture medium containing LPA were used as control to induce cytotoxicity. The evaluation of HUVEC was performed no earlier than 12 h after the exchange of the cell culture medium to avoid misinterpreting the endothelial cell morphological state.

### 2.2. Production of the AP Powder

AP used for cultivation was obtained from the “The Culture Collection of Algae at Goettingen University” (strain: SAG21.99). AP were cultured in a flat-type (2 cm) vertical transparent bioreactor consisting of a flexible polyethylene (PE, food-safe grade) sleeve with a 1.0 L working volume. The growth medium (Zarrouk medium [33] was initially sterilized at 121 °C in a HV-50 autoclave (SYSMEX VX-95, Sysmex, Norderstedt, Germany) for 15 min. The bioreactor was thermostatted to 30 °C and illuminated by fluorescent lamps. Magnetic stirrers were used for mixing [34]. Photon flux densities of 50 μE/(m^2^/s) were applied at a 24/0 h photoperiod using fluorescent lamps. The bioreactor was aerated with 0.236 vvm air with 2% CO_2_. 0.5 L/h 100% CO_2_ in 25 L/h atmospheric air were applied. The air was pumped through a membrane filter (Millipore; 0.45 μm pore size, 10 cm diameter) and moistened by passage through distilled water. Every second day of cultivation, optical density, dry weight, pH value, nitrate, and phosphate concentrations were measured. Phycocyanin, allophycocyanin, phycoerythrin, chlorophyll-a, carotenoids, and fatty acids were measured 28 days after inoculation.

The phycocyanin concentration was 108.5 + 1.5 µg/L, allophycocyanin 31.6 µg/L and phycoerythrin 32.9 µg/L. The concentration of chlorophyll-a was 12.1 + 3.9 mg/g, and carotenoid 9.0 + 7.1 mg/g. Elemental analysis yielded the following values: nitrogen content of about 10.5% and a sulfur content of about 0.6%. The difference in carbon was about 2%.

The protein content was 66.1 ± 0.18%.

The AP powder obtained was stirred overnight in a sterile 0.9% NaCl solution (B.Braun, Melsungen, Germany) at room temperature (10 mg/mL). The extracts were then centrifuged at 3400× *g* for five minutes, followed by sterile filtration using 0.4 and 0.22 µm filters (TPP). The extracts were stored at 4 °C until further processing.

### 2.3. Endothelial Cells

Primary human umbilical vein endothelial cells (HUVEC) were purchased from Lonza (Basel, Switzerland). HUVECs were cultivated under static conditions in a standard humidified incubator at 37 °C with 5% CO_2_ supplemented with 10% fetal calf serum (Thermo Fisher Scientific, Vienna, Austria; 10270106) and 100 U/mL penicillin/streptomycin. Cell experiments were performed using HUVEC in passage 4. Cell culture medium (Lonza, Basel, Switzerland) was changed every two days.

### 2.4. Secretion Profile Screening

The release of prostacyclin (PGl2) and thromboxane A2 (TXA2) was measured as a sign of HUVEC function two and four days after cell seeding using ELISA. The concentrations of PGl2 and TXA2 in the cell culture supernatant are detected using the 6-keto Prostaglandin F1α EIA Kit (Cayman Chemicals, Hamburg, Germany) and TXA2 Kit (Cloude-Clone Corp., Houston, TX, USA) in the cell culture supernatant.

### 2.5. Analysis of Cell Viability, Cell Growth and Cell Morphology

Cell viability was analyzed using fluorescein diacetate (FDA, 25 µg/mL Invitrogen, Carlsbad, CA, USA) to stain vital cells green and propidium iodide (PI, 2 µg/mL, Sigma, Taufkirchen, Germany) to stain dead cells red, two and four days after seeding. Three pictures per sample were then taken using 10-fold primary magnification in fluorescence and phase contrast microscopy (LSM 510 META, Zeiss, Oberkochen, Germany).

### 2.6. Immunofluorescence Staining

Two and four days after seeding the HUVECs on glass coverslips, the cells were washed with a phosphate-buffered saline solution, containing magnesium and calcium (PBS+/+; Biochrom GmbH, Berlin, Germany). Then, HUVEC were incubated with 4% paraformaldehyde solution (PFA; C. Roth, Karlsruhe, Germany). The HUVECs were washed with PBS without magnesium and calcium (PBS−/−; Sigma Aldrich, St. Louis, MO, USA). Permeabilization of the HUVECs was performed by incubating the cells with a solution of 0.25% Triton X-100 (VWR, Darmstadt, Germany). Then, the unspecific binding sites were blocked using a BSA solution (3% in PBS−/−, Biochrom GmbH, Berlin, Germany). vWF was stained using an anti-vWF polyclonal rabbit primary antibody (1:500 in PBS, Thermo Fisher Scientific, Waltham, MA, USA). VE-cadherin was stained using an anti-VE-Cadherin rabbit antibody (1:60 in PBS, Beijing, China). Phalloidin-iFluor 488 Reagent (1:500 in PBS with BSA, Abcam, Cambridge, UK) was used to stain the cytoskeleton. Anti -rabbit Alexa Fluor 555 (1:1000, Invitrogen, Carlsbad, CA, USA) was then used as a secondary antibody, together with 4′,6-diamidino-2-phenylindole (DAPI, Roth, Karlsruhe, Germany) to stain the nuclei. Finally, the HUVEC were subjected to a thorough wash with Phosphate-Buffered Saline (PBS), followed by a meticulous embedding process utilizing ProLong™ Gold Antifade Mountant (Invitrogen, Carlsbad, CA, USA).

### 2.7. Cell Membrane Integrity

The analyses of cell membranes were conducted by means of LDH-Cytotoxicity Assay Kit II© (BioVision Inc., Milpitas, CA, USA). The release of intracellular lactate dehydrogenase in cell culture supernatant was measured two and four days after cell seeding.

### 2.8. Analysis of Cell Metabolism

The metabolic activity of HUVEC was investigated using a one-step MTS test (CellTiter 96^®^ Aqueous Non-Radioactive Cell Proliferation Assay, Promega, Madison, WI, USA). In the context of this assay cytosolic NADH-dependent dehydrogenases of metabolic active cells are reducing the tetrazole group of added components to formazan. The amount of formazan is directly proportional to the number of cells.

### 2.9. Analysis of Interleukin-6

The analysis of proinflammatory IL-6 was conducted using human IL-6 ELISA Kit (Sigma Aldrich, St. Louis, MO, USA). The measurement was performed using cell culture supernatant two and four days after cell seeding.

### 2.10. Statistical Analysis

Means and standard deviations were calculated for all samples. Utilizing the data from a preceding pilot study, a sample size estimation was performed, which resulted in the determination of an optimal sample size of n = 8 experiments for all subsequent experiments.

One-Way Anova for paired samples was used to analyze significant differences. A *p* value of less than 0.05 was considered to be statistically significant.

### 2.11. AI Tool

The graphical abstract was created with assistance of generative AI (manus.ai, v. 1.6) using the Nano Banana Pro image generation tool. The AI was only used to generate a visual summary based on the authors’ original manuscript and experimental figures. The authors have reviewed and approved the graphical abstract and take full responsibility for its scientific accuracy.

## 3. Results

### 3.1. Effect of Arthrospira platensis on the Growth of Endothelial Cells with Lipopolysaccharides

**Figure 1:**
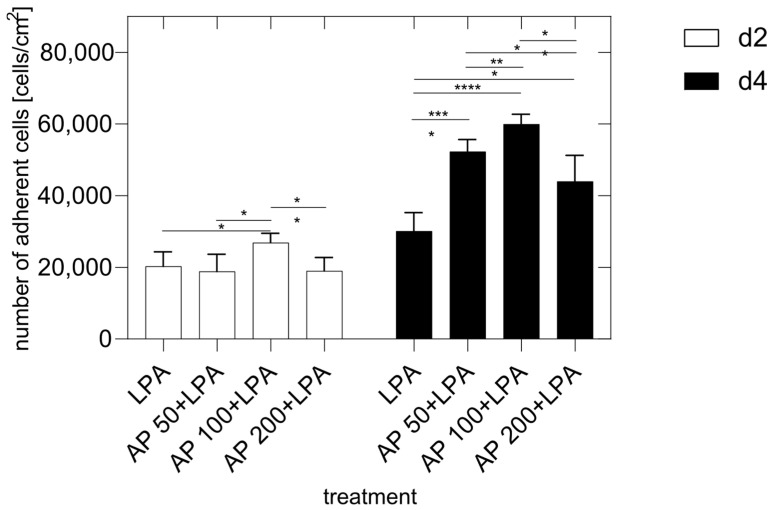
Number of adherent HUVEC per cm^2^ (vital and dead) 2 and 4 days after cell seeding. LPA: HUVEC in medium with 2.5 µg/mL LPA. AP 50 + LPA: HUVEC in 50 µg/mL AP extract + 2.5 µg/mL LPA, AP 100 + LPA: HUVEC in 100 µg/mL AP extract + 2.5 µg/mL LPA, AP 200 + LPA: HUVEC in 200 µg/mL AP extract + 2.5 µg/mL LPA. Significance levels: * = *p* ≤ 0.05, ** = *p* ≤ 0.01, *** = *p* ≤ 0.001, **** = *p* ≤ 0.0001. Means ± standard deviations (SD) are shown with n = 8 independent experiments.

In the control cultures, the HUVEC numbers/cm^2^ increased by 130.5% on day 4 (46,720 ± 6474) compared to day 2 (20,273 ± 3377). In contrast, the addition of LPA to the culture medium significantly reduced the growth of HUVEC compared to the growth of the control cells (day 2: 20,289 ± 4051, day 4: 30,149 ± 5167). Here, the increase in HUVEC on day 4 versus day 2 was only 48.6% (see Figure 1).

The addition of 100 µg/mL of AP to the cell culture medium of LPA-treated HUVEC led to a significant increase in the HUVEC cell numbers compared to the LPA-treated cultures after two days (*p* < 0.05). On the other hand, the addition of 50 µg/mL or 200 µg/mL of AP had no influence (see Figure 1).

**Figure 2:**
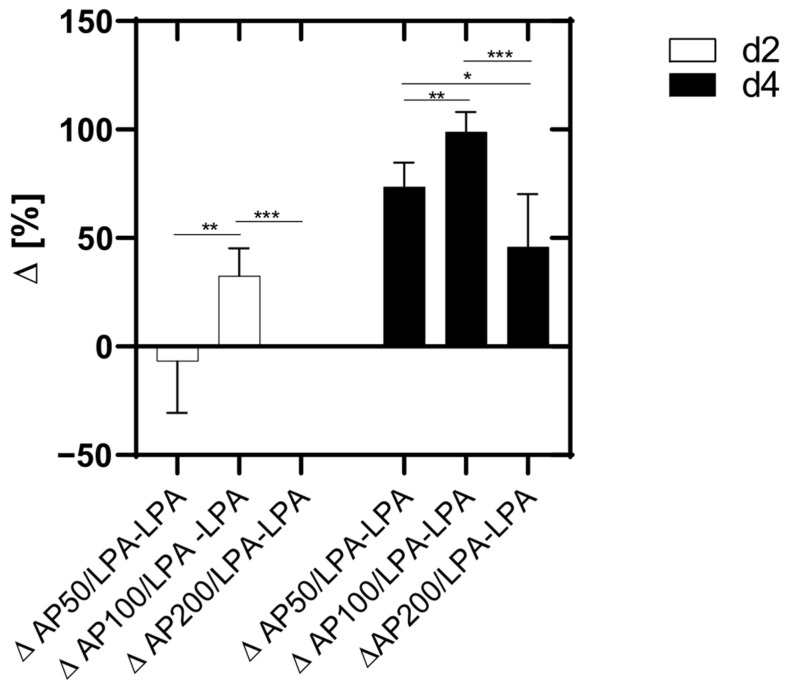
Differences between the growth of cells of AP50, AP100, AP200/LPA-treated cells versus LPA-treated HUVEC. Significance levels: * = *p* ≤ 0.05, ** = *p* ≤ 0.01, *** = *p* ≤ 0.001. Means ± standard deviations (SD) are shown with n = 8 independent experiments.

Four days after cell seeding a significantly higher number of adherent HUVEC was seen for cells cultivated with AP 50/100/200 compared to LPA alone. The LPA treatment of HUVEC decreased the number of adherent cells significantly. This effect of LPA and AP extract is also shown in Figure 2. Here the positive effect of AP extract supplementation to LPA-treated cultures can be observed by differences between adherent HUVEC of AP/LPA versus LPA-treated cells. This diagram clearly shows that the AP-supplementation of the cell culture medium significantly diminished the negative effect of LPA on cell growth and cell proliferation 4 days after cell seeding dose dependently (AP 50/LPA: 73.6% ± 11.2; AP 100/LPA: 98.9% ± 9.1%; AP 200/LPA: 46% ± 24.2).

**Figure 3:**
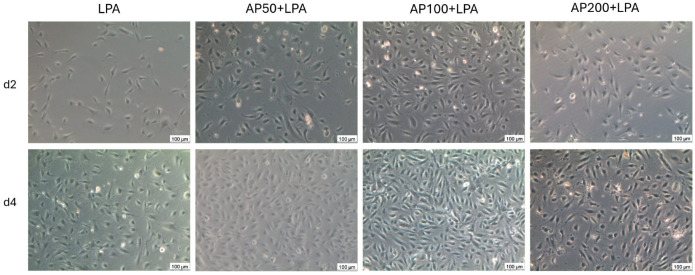
Representative images of cell cultures at day 2 (**upper row**) and day 4 (**lower row**) for the LPA-treated HUVEC as well as for the AP-treated HUVEC. Pictures were taken by phase contrast microscopy in transmission with 10× primary magnification. (scale bar: 100 µm).

Thus, it can be summarized that LPA treatment of HUVEC affected cell adherence and/or cell proliferation negatively whereas the supplementation of cell culture medium with LPA diminished this effect significantly (see Figure 3).

The LPA treatment of HUVEC affected cell adherence and/or cell proliferation negatively whereas the supplementation of cell culture medium with AP100 diminished this effect significantly. The images are in close correlation to the cell density measurements in Figure 1. The highest HUVEC density can be seen for the culture supplemented with AP100, the lowest one for the HUVEC culture supplemented with LPA but without AP.

**Figure 4:**
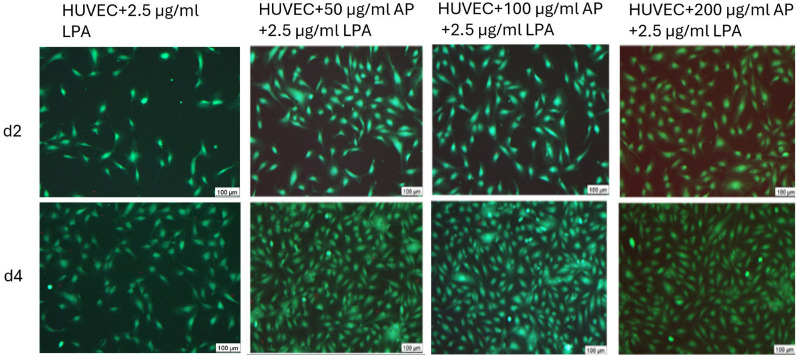
Representative images of fluorescence-based detection of live and dead cells under all tested conditions two and four days after cell seeding. (green: living HUVEC, red: dead HUVEC).

Also, a further independent method, the staining of live and dead cells, showed similar results. Figure 4 shows an example of the staining of vital cells (green) and dead cells (red) two and four days after cell cultivation. As expected, the images show a clear increase in the number of cells over the duration of cultivation. Furthermore, it can be seen that only a few dead cells can be recognized (Figure 4).

### 3.2. Impact of AP Extract on the Viability of LPA-Harmed HUVECs

**Figure 5:**
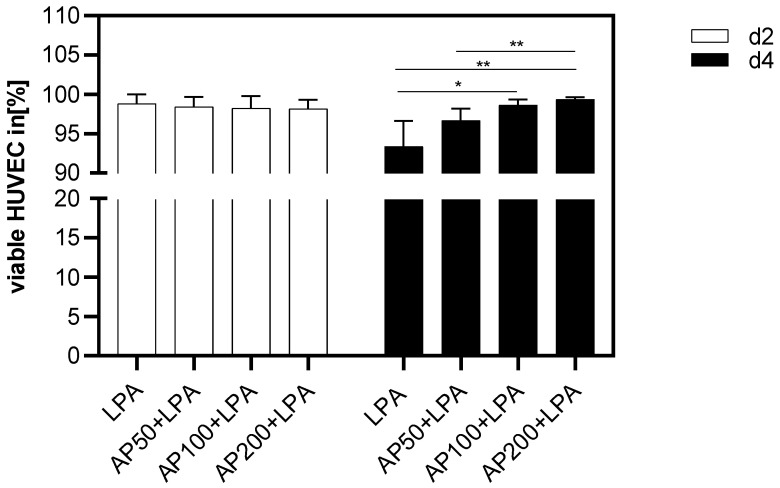
Viability of HUVEC in % two and four days after cell seeding. HC: high control: HUVEC lysed with Triton, LPA: HUVEC in medium with 2.5 µg/mL LPA. AP50 + LPA: HUVEC in 50 µg/mL AP extract + 2.5 µg/mL LPA, AP100 + LPA: HUVEC in 100 µg/mL AP extract + 2.5 µg/mL LPA, AP200 + LPA: HUVEC in 200 µg/mL AP extract + 2.5 µg/mL LPA. Significance levels: * = *p* ≤ 0.05, ** = *p* ≤ 0.01. Means ± standard deviations (SD) are shown for n = 8 independent experiments.

Figure 5 shows the quantitative analysis of the cell viability two and four days after HUVEC seeding. Two days after cell seeding, no significant differences could be found between LPA- and LPA/AP-treated HUVEC and AP extract-treated HUVEC in terms of cell vitality.

Four days after cell seeding a significantly increased cell viability was observed for AP50, AP100 and AP200 treated cells compared to LPA-harmed HUVEC. Within the AP-treated group a significantly higher viability was seen for AP200 cultivated cells compared to AP50 (Figure 5).

### 3.3. Impact of AP Extract on Cell Membrane Integrity of LPA-Harmed HUVECs

To investigate the influence of LPA and AP extract on cell membrane integrity the release of lactate dehydrogenase, an intracellular enzyme, in the cell culture supernatant was analyzed. A high release of this enzyme is associated with an eminent damage of the cell membrane.

**Figure 6:**
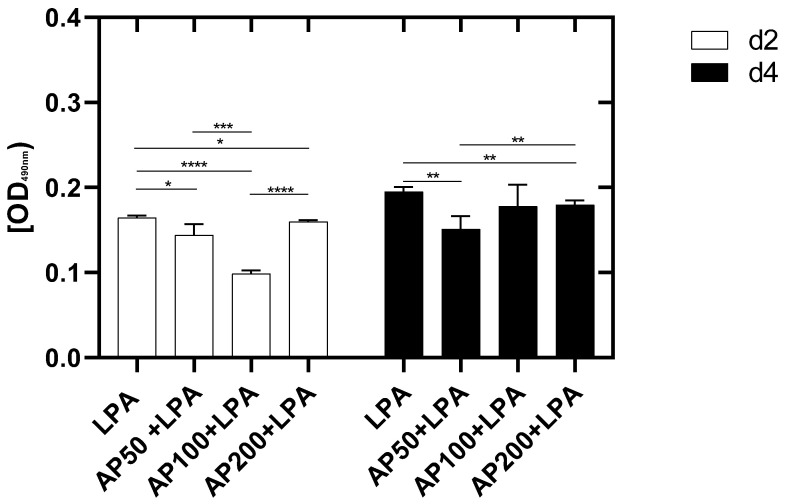
Cell membrane integrity of HUVEC 2 and 4 days after cell seeding. The activity of released LDH was measured as indicator of cell damage. HC: high control: HUVEC lysed with Triton, LPA: HUVEC in medium with 2.5 µg/mL LPA. AP50 + LPA: HUVEC in 50 µg/mL AP extract + 2.5 µg/mL LPA, AP100 + LPA: HUVEC in 100 µg/mL AP extract + 2.5 µg/mL LPA, AP200 + LPA: HUVEC in 200 µg/mL AP extract + 2.5 µg/mL LPA. Significance levels: * = *p* ≤ 0.05, ** = *p* ≤ 0.01, *** = *p* ≤ 0.001, **** = *p* ≤ 0.0001. Means ± standard deviations (SD) are shown with n = 8 independent experiments.

The analysis of the cell membrane integrity showed a significant increased release of LDH for endothelial cells cultivated with LPA compared to HUVEC which were additionally cultivated with AP50, AP100 and AP200. Thus, the cultivation of primary endothelial cells with LPA had a significant influence on the integrity of the HUVEC membrane. In addition, it was observed that the lowest LDH-release occurred for HUVEC treated with AP100/LPA compared to AP50 and AP200 (Figure 6).

Four days after cell seeding a significantly increased LDH-release was detected for LPA-treated HUVEC compared to HUVEC, which were cultivated with AP50/LPA and AP200/LPA.

In conclusion, the supplementation of HUVEC cell culture medium with AP extract had a protective effect on cell membrane integrity, which was negatively affected by LPA-treatment (Figure 6).

### 3.4. Influence of AP Extract on the Metabolic Activity of LPA-Harmed HUVEC

**Figure 7:**
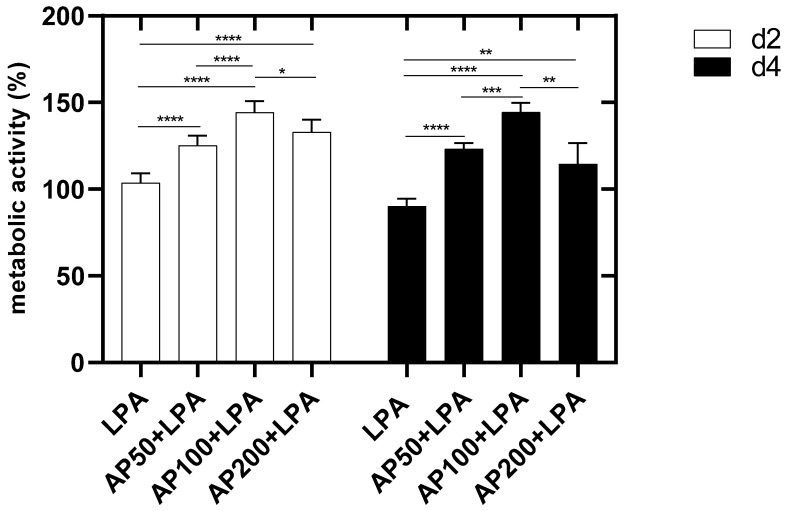
Metabolic activity of HUVEC two and four days after cell seeding. HC: high control: HUVEC lysed with Triton, LPA: HUVEC in medium with 2.5 µg/mL LPA. AP50 + LPA: HUVEC in 50 µg/mL AP extract + 2.5 µg/mL LPA, AP100 + LPA: HUVEC in 100 µg/mL AP extract + 2.5 µg/mL LPA, AP200 + LPA: HUVEC in 200 µg/mL AP extract + 2.5 µg/mL LPA. Significance levels: * = *p* ≤ 0.05, ** = *p* ≤ 0.01, *** = *p* ≤ 0.001, **** = *p* ≤ 0.0001. Means ± standard deviations (SD) are shown with n = 8 independent experiments.

Two days after cell seeding a metabolic activity of 103.7% ± 5.6% was observed for LPA-treated HUVEC. Furthermore, for all cell cultures a metabolic activity of more than 100% was seen (Figure 7). The metabolic activity of HUVEC only treated with LPA had a significantly lower metabolic activity compared to HUVEC cultivated with additional AP extract (AP50, AP100, AP200). Therefore, it can be concluded that the cultivation of cells with AP extract had a significant influence on cell metabolism. Nevertheless, a harmful component was added and cell metabolism was improved by cultivating HUVEC in AP extract supplement medium. In addition, HUVEC seeded in AP100/LPA exhibited a significantly higher metabolic activity compared to HUVEC in AP50/LPA and AP200/LPA (Figure 7).

Four days after cell seeding the metabolic activity of LPA-treated HUVEC declined to 90.2% ± 4.4%. The cultivation of HUVEC cultivated with additional AP extract (AP50, AP100, AP200) induced a significant increase in the metabolic activity compared to LPA-treated cells. It was also seen here that the highest metabolic activity was induced by supplementation of AP100 (Figure 7).

### 3.5. Analysis of HUVEC Function

#### 3.5.1. Release of Prostacyclin (PGl2)

Prostacyclin (PGl2) which is released by the endothelium to control vessel tone and platelet aggregation is a marker of cell function [11].

**Figure 8:**
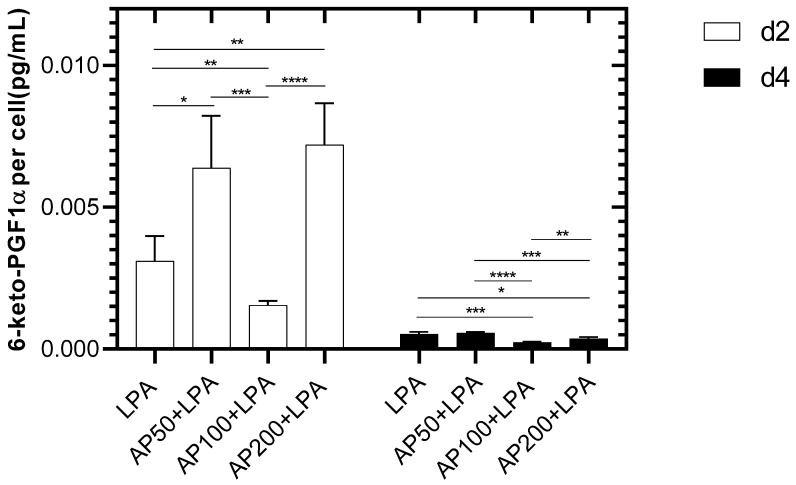
PGl2 secretion of HUVEC two and four days after cell seeding. LPA: HUVEC in medium with 2.5 µg/mL LPA. AP50 + LPA: HUVEC in 50 µg/mL AP extract + 2.5 µg/mL LPA, AP100 + LPA: HUVEC in 100 µg/mL AP extract + 2.5 µg/mL LPA, AP200 + LPA: HUVEC in 200 µg/mL AP extract + 2.5 µg/mL LPA. Significance levels: * = *p* ≤ 0.05, ** = *p* ≤ 0.01, *** = *p* ≤ 0.001, **** = *p* ≤ 0.0001. Means ± standard deviations (SD) are shown with n = 8 independent experiments.

A significantly reduced PGl2 release was observed for AP100/LPA-treated HUVEC two days after seeding compared to cells cultivated with LPA-, AP50/LPA- and AP200/LPA-cultivated HUVECs (Figure 8). The highest release was seen for HUVEC treated with AP200/LPA.

The release of PGl2 was diminished for all samples four days after cell seeding. Here, comparable to the data, after two days the lowest release was seen for HUVEC in AP100/LPA compared to AP50-, AP200 and LPA-treated cells (Figure 8). In addition, a lower PGl2 release was observed for HUVEC cultivated in AP200/LPA compared to LPA- and AP50/LPA-treated cells So, it can be summarized that the highest PGl2 release was seen 2 days after seeding, but declined after four days. Therefore, this AP extract concentration seems to have the biggest influence on the cells. Interestingly, this influence decreased drastically after four days.

#### 3.5.2. Release of Interleukin 6 (IL-6)

**Figure 9:**
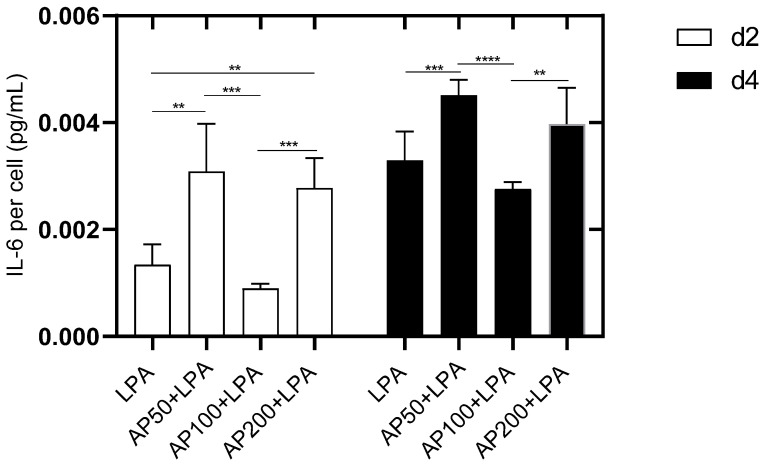
Presentation of the IL-6 secretion of HUVEC two and four days after cell seeding. LPS: HUVEC in medium with 2.5 µg/mL LPA. AP50 + LPS: HUVEC in 50 µg/mL AP extract + 2.5 µg/mL LPA, AP100 + LPA: HUVEC in 100 µg/mL AP extract + 2.5 µg/mL LPA, AP200 + LPA: HUVEC in 200 µg/mL AP extract + 2.5 µg/mL LPA. Significance levels: ** = *p* ≤ 0.01, *** = *p* ≤ 0.001, **** = *p* ≤ 0.0001. Means ± standard deviations (SD) are shown with n = 8 independent experiments.

In order to investigate whether endothelial cells are activated by AP extract the release of interleukin 6 (IL-6) as proinflammatory cytokine was analyzed. Two days after cell seeding a significantly lower IL-6 release was measured for HUVEC treated with LPA alone compared to HUVEC with AP50/LPA and AP200/LPA. Within the AP-treated groups the significantly diminished release was observed for HUVEC cultivated with AP100/LPA (Figure 9).

An increased release of proinflammatory IL-6 was seen for all HUVEC cultures four days after cell seeding. In trend the lowest release was measured for AP100/LPA cultivated cells compared to the other LPA- and AP-cultivated HUVEC. IL-6 by LPA-treated HUVEC was significantly lower than AP50/LPA-treated HUVEC but comparable to AP200-cultivated cells. In trend, the highest IL-6 release was observed for AP50-treated cells. Therefore, it can be concluded that the cultivation of HUVEC with AP extract induces a proinflammatory activation. This proinflammatory activation was partly diminished for HUVEC treated with LPA alone.

Furthermore, it became obvious that a cultivation of HUVEC with AP100/LPA reduced the cytokine significantly. It seems that this extract concentration has anti-inflammatory effects (Figure 9).

#### 3.5.3. Thromboxane A2

Thromboxane (TXA2) is a prostacyclin antagonist. Thus, TXA2 also has an influence on the regulation of the blood vessel diameter and therefore on blood flow and blood pressure. The release of TXA2 induces an increase in the blood vessel tone and thus a narrowing of blood vessels. Furthermore, the eicosanoid promotes thrombocyte activation and aggregation.

Interestingly, no TXA2 was detected in the supernatant of HUVEC. There are several possible reasons for this result: TXA2 was not released, or the amount of TXA2 released was below the detection limit of 15.625 pg/mL of the assay used.

### 3.6. Immunofluorescence Staining

In addition, the cells were immune-fluorescently stained two and four days after fixation with paraformaldehyde (PFA). This involved three staining steps: (i.) of the actin filament, (ii.) the cell nuclei and (III.) the von Willebrand factor. In the first step, the cytoskeleton (actin filaments), which is responsible for maintaining the cell shape and contact with the substrate, was stained. The formation of stress fibers can be seen as a sign of a not yet functionally confluent endothelial cell monolayer, but also as an adaptation to external conditions such as shear forces or substances [35]. The cell nuclei were then stained in order to assess the cells and their expansion. In addition, an endothelial cell-specific marker was stained for identification of the HUVEC (von Willebrand factor, vWF). In vivo, vascular endothelial cells are exposed to strong shear stress due to the permanent blood flow. Thus, the attachment of the cells to the underlying substrate and the formation of cell–cell contacts should not be negatively influenced by the addition of the AP extract.

**Figure 10:**
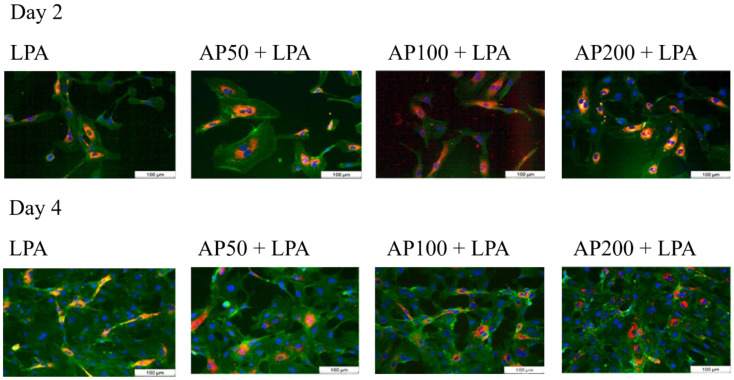
Representative images of HUVEC monolayers at days 2 and 4 after seeding (actin cytoskeleton (green), von Willebrand factor (red) and genomic DNA (blue) fluorescently stained. Shown are HUVEC of cultures with the addition of LPA, Arthrospira50 extract + LPA, Arthrospira100 extract + LPA, Arthrospira200 extract + LPA. (laser scanning microscope, LSM 510 META, Zeiss, Oberkochen, Germany).

The microscopic examination of the cells showed that the cells used were endothelial cells, as expected, due to the staining of the vWF in red. This clear characterization was necessary, particularly with regard to the interpretation of the results. Furthermore, it became clear at the time of examination in all samples that all cells still contained stress fibers after 4 days. Stress fibers are preferentially present inside the cell and are a clear sign that a functional–confluent monolayer is not yet present. This state of the endothelial cells is known and described as perturbation. It is a typical reaction to changes in the cell environment (e.g., new culture medium, drugs, contrast agents, etc.). A reorganization of the stress fibers into the cell rims was already visible for the cultures supplemented with AP100 at day four (Figure 10).

In addition, vinculin and VE-cadherin were also stained. Vinculin is a typical cell marker that primarily causes the establishment of cell–substrate contacts. The distribution and expression of this protein can therefore also be used to draw conclusions about the binding of endothelial cells to the underlying substrate. VE-cadherin (vascular endothelial cadherin) is a protein that occurs in contact sites of neighboring endothelial cells. This protein is therefore essential for the maintenance and control of endothelial cell contacts and thus for the control of vascular permeability and leukocyte extravasation, among other things.

Two or four days after cell seeding, all cells (with and without the addition of AP or LPA) showed uniform vinculin expression. The formation of cell–cell and cell–substrate contacts was thus not impaired despite the addition of LPA or the AP extract.

**Figure 11:**
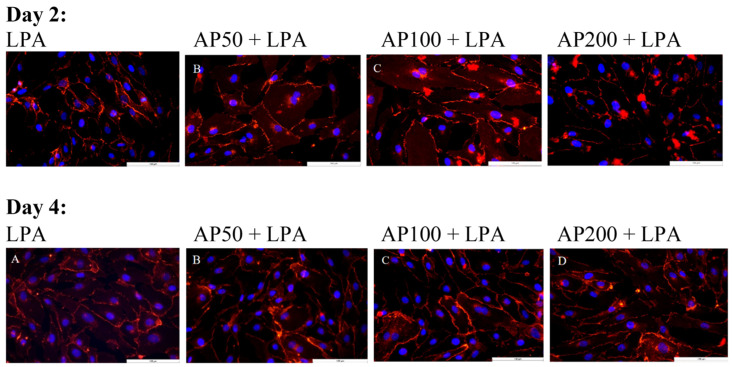
Cell–cell contact marker VE-cadherin two and four days after seeding (VE-cadherin: red, genomic DNA: blue) fluorescently stained. Shown are HUVEC cultures with the addition of LPA, Arthrospira50 extract + LPA, Arthrospira100 extract + LPA, Arthrospira200 extract + LPA. (laser scanning microscope, LSM 510 META, Zeiss, Oberkochen, Germany).

In contrast, HUVEC treated with 200 µg/mL AP extract showed an apparently enlarged cell morphology two days after cell seeding. Four days after cell seeding, this phenomenon was no longer observed. Two days after cell seeding, there was an increased presence of intracellular VE-cadherin in HUVEC treated with LPA. Four days after cell seeding, this could no longer be detected (Figure 11).

## 4. Discussion

The study revealed that LPA induced damage to the morphology and function of HUVECs. This damage could be significantly reduced by pre-treating the cell culture with AP.

Earlier in vitro studies have shown that endothelial cells can lose their antithrombotic capacity when activated by several factors present under pathological conditions [36]. These factors include tumor necrosis factor (TNFα) [37], tissue factor [38], endotoxins such as LPA [39,40], interleukin-1 [41], chemokines [42] and thrombin [43]. LPS has also been shown to damage HUVEC morphology [9,38,44] and, ultimately, lead to cell death via an NAD(P)H oxidase-dependent ROS generation mechanism [45]. Schöffel attributed the slight reduction in adherent HUVECs to a decrease in HUVEC proliferation [46]. It has been reported that LPS binding to Toll-like receptor 4 (TLR4) results in endothelial activation. This is associated with the expression of proinflammatory cytokines, HUVEC detachment, and in severe cases, endothelial apoptosis or even apoptosis [5,10,11,19,47]. The molecular mechanism of LPS-induced endothelial dysfunction leading to apoptosis has been extensively studied in recent years [10].

These in vitro studies were confirmed by a dose-dependent in vivo study with 2 mg/kg LPS administered intraperitoneally (i.p.), which demonstrated a maximal prothrombotic effect in 2.5% ferric chloride-induced vena cava thrombosis. Thrombus size increased by 60% (n = 8, *p* < 0.05) compared to vehicle treatment [21]. In our study, we investigated the effects of AP on the response of HUVEC to 2.5 µg LPA/mL, because AP has been described to possess antithrombotic properties [48,49]. Therefore, the main question of the study was to analyze whether AP is able to protect HUVEC from the deleterious effects of LPA, which can lead to the activation and detachment of HUVEC, a release response, and ultimately to apoptosis. Our results are well in line with previous studies showing that endotoxinemia can induce HUVEC activation and detachment [14,15,16].

Furthermore, the study clearly revealed a dose-dependent response of adherent HUVECs when the culture medium was supplemented with AP 24 h before the addition of LPA. After the addition of 100 µg/mL AP, the highest number of adherent HUVECs was found with 130.5% (from day 2 to day 4), whereas after the addition of LPA alone the increase in adherent HUVECs on day 4 versus day 2 was only 48.6% (*p* < 0.05). The addition of 50 mg/dL or 200 mg/dL of AP had no significant effect (see Figure 1).

**Figure 12:**
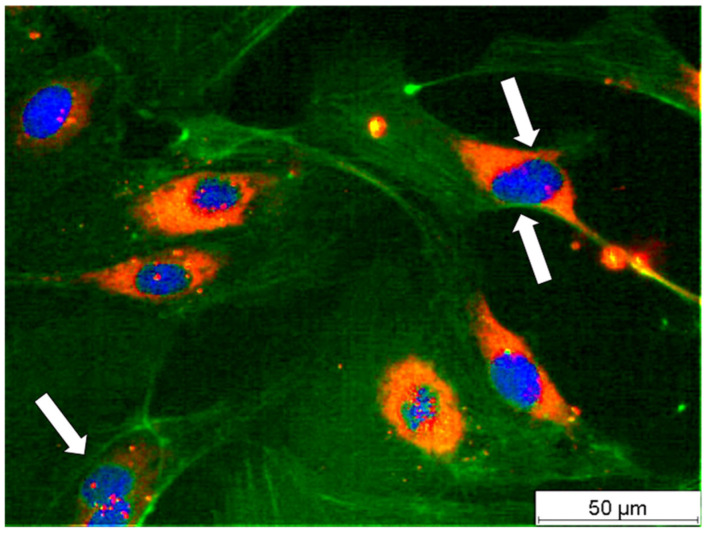
Triple-stained human umbilical vein endothelial cells (HUVEC) in a 24-well plate four days after seeding; actin cytoskeleton (green), von Willebrand factor (red) and genomic DNA (blue) using a widefield fluorescence microscope in 40× primary magnification.

The number of HUVECs per area were counted by live/dead staining, while the detection of von Willebrand factor allows the identification as endothelial cells. The detection of actin fibers within HUVECs indicated that the endothelial cells are still activated [37,50,51]. Figure 12 shows a triple-stained image of an almost confluent layer of HUVECs four days after seeding in a 24-well plate.

At this time, the HUVEC monolayer was not yet fully confluent and there were still gaps between the cells, as observed in previous endothelial cell (EC) studies [52]. This correlated well with the formation of stress fibers crossing through the cytoplasm, indicating that the HUVECs were motile and activated. In the lower left part of the image (see arrow), a dividing HUVEC can be seen with a distinctly ruffled nuclear border. A similar ruffled rim can also be seen to a lesser extent in the other HUVECs. Additionally, the early phase of nuclear division can be seen to the right at the top of the image (see double arrow). This demonstrates the relatively high mitotic activity and proliferation rate of the HUVECs at this stage. Some HUVECs already had a marginal filament band, while others were just beginning to form one.

By day four, VE-cadherin had begun to accumulate at the cell rims, establishing inter-endothelial junctions (see Figure 11). VE-cadherin is known to be the major determinant of endothelial cell–cell contact integrity [53], stabilizing the HUVEC monolayer. This was particularly evident in cultures supplemented with 100 µg/mL AP, but barely observable in cultures treated with LPA alone or with 50 µg/mL or 200 µg/mL AP prior to LPA administration.

Two or four days after seeding the cells, uniform vinculin expression was observed in all cells, with or without the addition of AP or LPA. Therefore, the formation of cell–substrate contacts did not appear to be affected by the addition of LPA or the AP extract for the adherent HUVEC.

Cell viability was significantly reduced for the cultures treated with LPA alone by day four as was shown earlier [45]. By contrast, the number of dead cells in the cell cultures to which AP had been added was significantly reduced at this time, with recovery increasing from 98.2 ± 1.6% to 98.6 ± 0.7% for the AP100 group on day four. The same was true of the integrity of the cell membrane, as evidenced by the reduced release of LDH. The same applied to the integrity of the cell membrane, recognizable by the reduced release of LDH. The metabolic activity of AP-treated HUVECs was higher than that of HUVECs supplemented with LPA alone. Once again, supplementation with AP100 induced the highest metabolic activity.

These findings are supported by the results of the release of prostacyclin, which is a potent vasodilator and inhibitor of platelet aggregation [54]. Activation of endothelial cells leads to an effect known as HUVEC perturbation [55]. In this state, endothelial cells induce cyclooxygenase-2, which is followed by an increase in prostacyclin generation. This coincides with the activation of the microfilament system in HUVECs and is accompanied by changes in cell-to-cell- or cell-to-substrate binding. This is characterized by a strong and rapid increase in prostacyclin (up to 40-fold above baseline levels [56]. The results of the present study now show that the initial supplementation of the culture medium with AP100 completely prevented an increase in prostacyclin following the administration of additional LPA (see Figure 8). The lack of perturbation of the endothelial monolayer correlates very well with the formation of the peripheral filament band and the reduction in stress fibers in central parts of the HUVEC. Similar findings were observed for IL-6, another proinflammatory interleukin, which also did not increase after the application of LPA when the culture medium was initially supplemented with AP100.

The impact of AP on HUVEC function is due to several intracellular responses. For example, AP induces the expression of endothelial nitric oxide synthase [57,58,59,60], which in turn increases NO production [61]. This increase in NO could stimulate HUVEC proliferation [62], which would be consistent with the results of the present study.

The anti-inflammatory effect of AP may also contribute to improved endothelial proliferation. AP has been shown to reduce both TnFα and TGFβ [63,64]. Reduced levels of TnFα or TGFβ concentration are described to inhibit the activation of adenine dinucleotide phosphate (NADPH) oxidase, thereby providing a potent antioxidant effect. This effect is discussed to prevent the overproduction of reactive oxygen species and thereby counteract apoptosis [65,66]. This could hypothetically explain the increased proliferation. Spirulina is considered to be easily digestible due to the absence of cellulose in its cell wall. There are a few studies on the digestibility of proteins and amino acids of Arthrospira. In a human study using the dual isotope method amino acid, the plasma appearance of amino acids and of free amino acids after oral intake of ASP was investigated [67]: AP amino acid digestibility ranged from 77.5% for lysine to 95.3% for phenylalanine with a mean indispensable amino acid digestibility of 85.2% [68]. Another study in rats using 15N intrinsically labeled AP found a nitrogen digestibility of 86.0 + 0.7% and of 83% of amino acids [69]. This shows that AP is very well absorbed. AP is partially digested in the gut, and constituents such as proteins and fats are absorbed through the intestinal mucosa in the form of amino acids, peptides and fatty acids. The aqueous extract also contains exopolysaccharides, which have been shown to exert an effect on cells. However, the extent to which and which components of the aqueous extract appear in the blood and thus in the body following oral administration remains largely unknown and still needs to be determined. In silico data indicate that phycocyanin has ‘excellent intestinal absorption’ and moderate plasma protein binding. However, it is considered highly likely that it is predominantly smaller phycocyanin fragments that circulate in the blood, rather than the complete protein [70]. Nevertheless, both animal models and human studies provide strong evidence that the intake of AP has a variety of health benefits and curative properties and is also capable of acting as an anti-inflammatory, antioxidant and, more recently, antiangiogenic agent [68].

## 5. Conclusions

In summary, the study confirmed that AP can minimize the damaging effect of LPA on HUVECs. The perturbation induced by LPA was completely prevented by 100 µg/mL AP, resulting in significantly faster development of the endothelial cell monolayer, accompanied by formation of a peripheral filament band and initial reduction in stress fibers in the center of the endothelial cells.

This was accompanied by a reduction in the cellular response to LPA (cell viability and cell membrane integrity), reduced cell metabolism (MTS assay) and reduced perturbation of HUVECs (reduction in PGI_2_ and IL-6). There was also reduced release of signaling molecules such as VE-cadherin and vinculin, which are important for stabilizing an endothelial cell monolayer and for cell–cell and cell–substrate binding.

These results clearly demonstrate that *Arthrospira platensis* has the potential to reduce damage to venous endothelium when exposed to lipopolysaccharides, particularly at a concentration of 100 µg/mL.

## Data Availability

The original contributions presented in this study are included in the article. Further inquiries can be directed to the corresponding author.

## Abbreviation

The following abbreviation is used in this manuscript:

LPA	Lipid A
